# Unconventional protein secretion (UPS): role in important diseases

**DOI:** 10.1186/s43556-022-00113-z

**Published:** 2023-01-09

**Authors:** Meldolesi Jacopo

**Affiliations:** 1 San Raffaele Institute, Vita-Salute San Raffaele University, Milan, Italy; 2 CNR Institute of Neuroscience at the Milano-Bicocca University, Milan, Italy

**Keywords:** Alzheimer’s, Cancer, Diseases, Ectosomes and exosomes, Endocytosis, Exocytosis, Diseases, Interconnected UPS, Membrane fusion, Neurodegeneration, Tau, Therapy, UPS = Unconventional Protein Secretion

## Abstract

Unconventional protein secretion (UPS) is the new secretion process discovered in liquid form over three decades ago. More recently, UPS has been shown to operate also in solid forms generated from four types of organelles: fractions of lysosomes and autophagy (APh) undergoing exocytosis; exosomes and ectosomes, with their extracellular vesicles (EVs). Recently many mechanisms and proteins of these solid forms have been shown to depend on UPS. An additional function of UPS is the regulation of diseases, often investigated separately from each other. In the present review, upon short presentation of UPS in healthy cells and organs, interest is focused on the mechanisms and development of diseases. The first reported are neurodegenerations, characterized by distinct properties. Additional diseases, including inflammasomes, inflammatory responses, glial effects and other diseases of various origin, are governed by proteins generated, directly or alternatively, by UPS. The diseases most intensely affected by UPS are various types of cancer, activated in most important processes: growth, proliferation and invasion, relapse, metastatic colonization, vascular leakiness, immunomodulation, chemoresistence. The therapy role of UPS diseases depends largely on exosomes. In addition to affecting neurodegenerative diseases, its special aim is the increased protection against cancer. Its immense relevance is due to intrinsic features, including low immunogenicity, biocompatibility, stability, and crossing of biological barriers. Exosomes, loaded with factors for pharmacological actions and target cell sensitivity, induce protection against various specific cancers. Further expansion of disease therapies is expected in the near future.

## Introduction

Secretion, a key function of all types of cells, was recognized to exist already in the first decades of the last century. At that time, however, the mechanisms of this function remained unknown. The integrated participation of intracellular organelles, i.e. the endoplasmic reticulum (ER) followed by the Golgi complex (GC) and then by secretory granules and vesicles discharged by exocytosis, was discovered in the cells of the exocrine pancreas [1] and then extended to other secretory cells [2, 3]. Additional studies discovered new processes about the steps by which secretory proteins are transferred across ER membranes [4] and then reach the GC [5]. More recently this process, considered unique, has been named and usually called conventional or canonical secretion.

In early 1990, however, distinct forms of another secretion started to emerge [6–9]. The unexpected, and thus unconventional protein secretion (UPS), was later recognized to operate by four different processes [10]. Types 1 and 2 of UPS [11, 12], typical of fluid proteins, were thought to depend on distinct agents of the plasma membrane including diffusion through lipids and binding/transport of molecules by surface channels and transporters [11–13]. The members of these two fluid types are numerous. At least some of them are able to operate by moving from their origin to target cells and vice-versa [14]. Fluid proteins, however, do not account for all UPSs. Studies started at the end of 1990, then extended and now widely accepted, demonstrate that two additional, 3 and 4 type secretions are solid components originated from specific intracellular organelles distinct from those of conventional secretion [15, 16]. Important properties of the four types of UPS in healthy cells and organs, initially proposed years ago [11, 15, 16], were confirmed according to general data and criteria [7, 8, 17, 18]. Recently, therefore, interest about UPS has increased considerably, approaching the levels existing since decades for conventional secretion.

Relevance of UPS, especially those of types 3 and 4, however, occurs not only in healthy cells, but also in many diseases. In the latter field many studies have been carried out, focused however, primarily or exclusively, on separate and distinct diseases (see for example [19–21]). Other recent UPS reviews included no diseases [7, 16] or only few details about them [17]. In my recent review diseases were lacking [18], however their general presentation was expected to appear in a coming article. In the recent literature, general view of UPS-dependent diseases had been reported only in two cases appeared in 2018 and 2019 [22, 23]. The quality of these two presentations is certainly considerable, however their focus is mostly different from that of the present work. The review by Kim et al. reported a wide view of all the 1–4 different, previously identified types of UPS. Because of its wide distribution and its lack of recent literature the coincidence of the Kim’s review with mine is limited. In the second general review, by New et al., the interest was focused primarily on autophagy (APh), with limited interest for the other mechanisms of UPS. In both previous reviews, diseases and therapy appeared only to minor levels [22, 23]. It appears, therefore, that my present review does not duplicate but expands areas of the field, including aspects and tools that had not been published previously.

Here the type 3 and 4 UPS diseases have been conceived by three general criteria. First, the presented data are focused on extensively investigated diseases. Second, the information provided appears valid because demonstrated or confirmed recently, i.e. during the last few years. Finally, the strategy of this review includes three Sections. The first is preparative, i.e. it illustrates in short the knowledge about UPS in healthy cells and organs, necessary for the subsequent presentation of diseases. The two following Sections are focused on the actions of UPS organelles, in particular of abundant exosomes, dealing with diseases of various types, including neurodegenerative, immune and cancer diseases, and their therapies.

## Role of UPS in healthy cells and organs

The present Section illustrates in short the available information about the UPS healthy systems, an approach that in the literature is often defined as physiology. In the present review I follow the latter definition. The task is to provide knowledge and understanding at least useful for the presentation of diseases and their therapy appearing in the subsequent Sections. In case of UPS details lacking here, they can be easily found in other reviews, appeared during the last few years [8, 16–18].

### Intracellular organelles involved in solid UPS

**Fig. 1:**
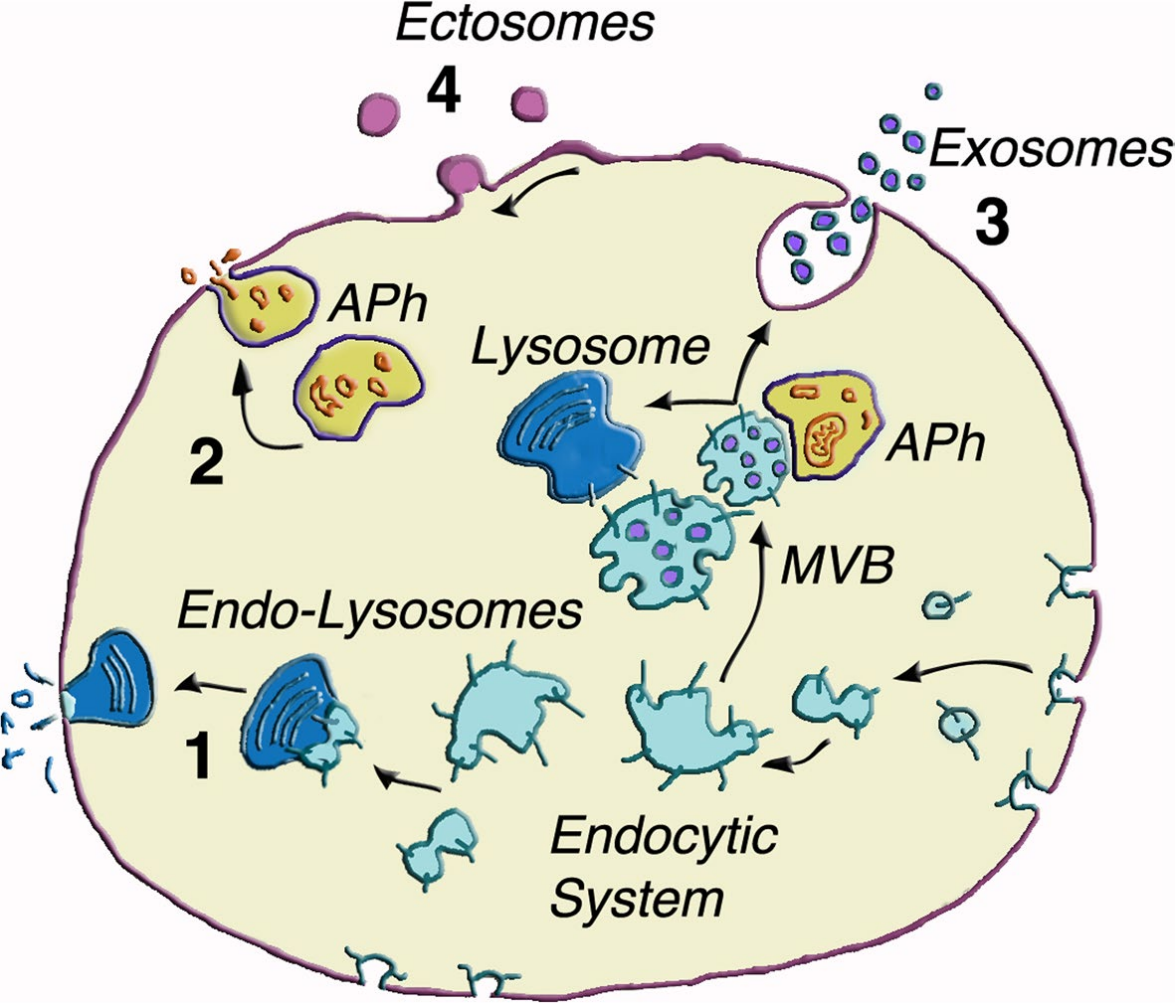
Cell showing the four UPS examples of exocytosis and vesicle release. Various UPSs are illustrated here as an introduction discussed later in the text. The Endocytic System (cerulean color) shows continuous uptake of small endoctic vesicles assembled into cisternae, some of which fusing with Lysosomes (1, dark blue). In addition to its digestive role the Endo-Lysosomes acquire their exocytic discharge [24, 25]. Autophagosomes (APh) (2, chamois yellow color, with candy cargo segregated and then discharged) interacts with endocytic multivesicular bodies (MVBs, cerulean color), relevant because of the abundance of their exosome components [26, 27]. Additional exchanges, taking place between the two types of EVs, occur after organelle exocytosis. Exosomes (3, violet vesicles) are highly specialized for discharge upon MVB exocytosis. In many cells they are abundant and play important roles in cancer development [30–35]. Ectosomes (4, amethyst color) are different from exosomes by size and generation. They include some components similar to those of exosomes.. This Figure is permitted as a modified version of Fig. 1 from [29]

The organelles of four distinct nature, active in the 3 and 4 types of UPS, exhibit structures and functions largely dependent on the specificity of their membranes. In view of the relevance of their properties, short presentation of these organelles are shown here (Fig. 1).

1. Lysosomes are cytoplasmic organelles specialized in their intracellular fusion with various types of organelles. Until a few decades ago, fusion with active lysosomes was believed to be direct, resulting in the digestion of discharged molecules. Beginning on early 1990 it become clear that, in order to proceed with their fusion activity, lysosomes need to receive interactions with endocytic structures (vesicles, vacuoles) of various age. Such activity, however, is not homogeneous. In a minor fraction lysosomes are more active and involve early endocytic components. Upon fusion these endo-lysosomes, characterized by specific markers [24], undergo regulated exocytosis, a function absent in the other forms of lysosomes [14, 15, 24, 25]. In this case, the discharge of lysosomal cargos to the extracellular space is variable. Together with typical molecules with specific enzymes it includes membrane fragments, molecules of various type and assembly, as well as vesicles of variable size, often considered among the extracellular vesicles (EVs).
2. APh is an organelle assembled by sequestration of cytosolic organelles and cargos within double membrane structures growing in the cytoplasm. Analogous to the other organelles, many mature APhs undergo fusion with lysosomes followed by degradation of their cargos. Growing evidence, however, has demonstrated that a fraction of them establish contacts with multivesicular bodies (MVBs, the organelles of exosome origin), important for the function of both [26, 27]. In contrast, other APhs undergo exocytosis. In the latter cases interactions can be established between EVs of this and other origin. By encompassing a wide range of secreted factors, APh secretion has been shown to induce many processes including toxic protein disposal, immune signaling and pathogen surveillance [25–27].
3. MVBs, vacuoles of endosomal nature. Upon activation of their ESCRT system, MVBs induce specialized generation and accumulation of many small vesicles (diameter of 50–150 nm), loaded with variable cargos. While in the cytoplasm these mature organelles can either fuse with lysosomes with ensuing digestion of their cargoes, or undergo stimulated exocytosis with ensuing release of their exosomes, into EVs [28, 29].
4. Ectosomes are larger vesicles (diameter of 150–400 nm), also called by other names (microvesicles, microparticles, shedding vesicles). They are directly generated in response to adequate stimulation by outward budding and fission of specialized plasma membrane microdomains [28, 29].

As already mentioned, exocytosis by membrane fusion with the plasma membrane releases EVs from the 1–3 organelles of the present paragraphs and Fig. 1. In this process the vesicular R-SNARE protein most frequently involved is VAMP7, which in contrast is inactive or marginal in the exocytosis of other organelles [17, 18]. The processes of endo-lysosomes and APhs are rare whereas those of exosomes are much more frequent. Such difference is among those accounting for the predominant role of the latter agent in UPS diseases, shown in the next Sections. However, the activity of MVBs and APhs is not always independent. During their intracellular traffic they may interact to each other (Fig. 1), with ensuing exchange of membrane components and also of cargo proteins. These processes are important for the following exocytosis an subsequent events of both MVBs and APhs [26, 27, 36]. In other cases, pre-fusion of APhs with endosomes participates in the generation of peculiar endo-autophagy organelles, often called amphisomes, which undergo frequent exocytoses. To cover other functions, however, also amphisomes undergo intracellular fusion with lysosomes [37].

Among the four organelles of UPS, ectosomes (number 4 in Fig. 1) have nothing to do with exocytosis. In fact they are generated by direct outward budding of plasma membrane microdomains. The ectosome generation is triggered by cdc42, a small G protein, and its effector, IQGAP1, together with additional G proteins, RhoA and Rock. ESCRT III, together with its ATPase VPS, account for the curvature of these EVs [38]. The direct ectosome vesicles, containing their luminal cargos, are released shortly after the start of their generation [29].

### Activity of EVs: from Navigation to cell targeting

Well-known products specifically released by conventional secretory cells are fluid proteins. Upon their release these proteins bind their receptors, often distributed in the proximity of the sites of their secretion. Compared to the general nature of the conventional products, the corresponding UPS products are more variable. Lysosomes and APhs have been shown to release many distinct molecules, for example interleukins, growth factors, enzymes and many others. Each of these molecules has specific functions. For example, most released enzymes govern the digestion of extracellular molecules [39]. In addition to molecules, release of some vesicles by exocytosis from lysosome and APhs cannot be excluded. In some studies, vesicles of this type have been reported [24, 38, 39].

On the other hand, the predominant release from exosomes and ectosomes is due to EVs, different from each other especially in size. Within such vesicles segregated luminal cargos contain, in addition to specific UPS proteins, also other specific proteins, lipids, various types of RNA and, in some cases, also small DNA sequences, surrounded by peculiar membranes [40, 41]. During EV navigation such vesicles resist long, up to months, even in aggressive environments. These membranes are therefore protective of the structure and composition of navigating exosomes and ectosomes [29, 42, 43]. Within EVs, exosomes are the most abundant. In specific studies, however, exosomes and ectosomes have been found present at similar levels [44, 45].

Exosomes released from single MVBs are frequently homogeneous. Heterogeneity of EVs, released by single or various types of cells and revealed by specific analyses [46–48], can occur during navigation and interaction with target cells. During navigation the EVs often move out their space by crossing of the blood–brain-barrier. As a consequence the EVs, even when generated in the brain, are spread to fluids of the body, including blood serum and cerebrospinal fluid. Specific binding of EVs to their target cells occurs by binding to receptors and fusion to plasma or endocytic membranes, followed by release of their cargos [45]. Functionally, therefore, signaling between cells takes place by both EV release and target cells. The latter receive and accumulate EV components.

### Unconventional secretion

Our focus deals with the 3 and 4 types of UPS [11, 12]. Most proteins secreted by these types lack any membrane spanning domains. Therefore, they cannot cross the ER membrane. This makes impossible their conventional secretion, which is replaced by fluid and solid forms of UPS. Proteins initially accumulated by organelles of the 3 or 4 types are numerous [17, 18]. Moreover, the identification of their markers is important [47, 48]. IL cytokines (including IL-1β, the key tool of UPS discovery [8, 9]) has already been mentioned; SCAMP5 is an inhibitor of APh fusion with lysosomes [49]; tau is a phosphoprotein which navigates by three UPS pathways, accumulating in the somato-dendritic and extracellular compartments. By propagating from cell to cell, tau is critical in various neurodegenerative diseases [50, 51]. UPS proteins of type 4 are able to cross the ER membrane, however, their conventional pathway is often blocked and replaced. Among these proteins is FADD, a controller of cell death, proliferation and other processes [52]; the pro-inflammatory transmembrane TACE [53]; α-synuclein, addressed to various forms of EVs [54]. Golgi proteins such as GRASP55, operate by conventional secretion when phosphorylated and active by UPS upon dephosphorylation [55]; proteins of the TMED complex are able to spun the ER membrane but are unable to move to the GC. They therefore move to a form of UPS associated to ER stress. By this approach relevant proteins, such as HMGB1 [25], CFTR, pendrin and SARS-CoV2 [56], are released. Additional examples of UPS can be found in [18].

## Role of UPS in diseases

The general introduction of UPS physiology has already illustrated many of the processes generated in several types of cells upon their activation. At this point the discussion can be extended to numerous diseases mechanistically governed by UPS. Activation/inhibition of such diseases can be induced by factors/events triggered in the cells, for example by ATP, stress, or exosomes. The ensuing processes induced in the cells include pathogenesis, diagnosis and regeneration, protection [57, 58], up to secretion and therapy (50). Our present interest about them is not only descriptive but also operational. In fact, knowledge about present UPS processes in various diseases can open perspectives for their future investigation.

### Neurodegenerative diseases

**Fig. 2:**
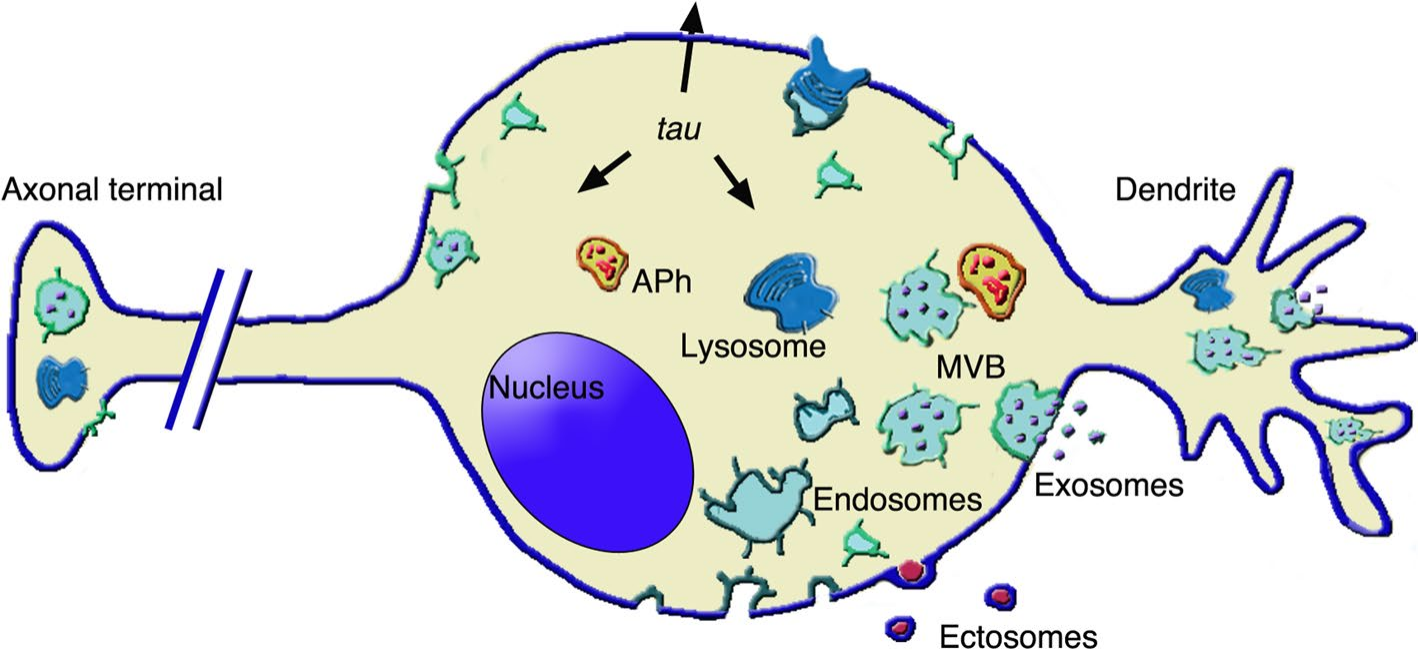
Neuron: intense circulation of MVBs and other organelles involved in UPS processes. The continuous uptake of endosome vesicles (cerulean color) leads to various cisternae, with some (MVBs) accumulating large numbers of vesicles (violet). MVBs circulate inside the cell, distributed not only in the cytoplasm but also in dendrites (right) and axon terminals (left). Exocytosis of MVBs (right) results in the extracellular discharge of exosomes. The Figure illustrates also other organelles with role in UPS. Lysosomes (dark blue), in addition to their well-known digestive functions, become competent for exocytosis (top) upon fusion with endosomes. Autophagosomes (APhs, chamois yellow color with candy segregated structures) also circulate in the cytoplasm. They interact with MVBs and then undergo parallel exocytoses. Ectosomes (bottom, amethyst) are released by budding and fission of plasma membrane microdomains. Finally the Figure illustrates the state in neurons of protein tau (three small arrows), secreted by various, fluid and solid UPS processes [62–66]

Brain alterations are highly important in the pathology of Alzheimer’s (AD), Parkinson’s (PD), and other neurodegenerative diseases. In neurons (Fig. 2), various aspects of disease deal with the control of protein misfolding within cargos of EV lumen [59, 60]. Biomarkers of these and other vesicles, involved in clinical diseases, include endosomes, endo-lysosomes [61] and APhs [27]. In these cases various stages of the diseases involve exosomes, released not only by neuron cell bodies but also by dendrites and axon terminals (Fig. 2) [57–59].

The predominant role in all the UPS effects reported here is predominantly depends on exosomes. This is due, on the one hand, to the abundance of their release by neurons (Fig. 2) and astrocytes; on the other hand, to the dynamics, the permeability and the persistence of their functions. Illustration of the numerous exosome activities would occupy too much space. To provide significant examples I have made a choice, including unconventional secretion of misfolded proteins taking place in a primary step of exosome growth, that of MVBs, together with diagnoses and protections, the last including the state of synapses (Table 1). Additional information about exosomes can be found in the present Section and previous publications [19, 20, 29, 36, 42].

**Table 1:** Key roles of exosomes in neurodegenerative diseases

	**Secretion**	**Diagnosis**	**Protection**	**Reference**
**MVBs**	Cytopl. Misfolded proteins.			[67, 68]
**EVs**		Biomarkers; Carriers of membrane cross.	Loading cargos; Product. Techniques; MSC traffic.	[57, 59, 60]
**Synapses**			Formation; Modulation of NTRs.	[69]

Exosomes play many critical roles in neurodegenerative diseases. Among such roles I have chosen three reported in the present Table. Misfolded proteins of cytoplasmic nature, relavant for unconventional Secretion, are loaded into exosome cargos during vesicle assembly at the MVB surface. EVs of exosomes, released upon MVB exocytoses, predominate in various types of functions: in terms of Diagnosis, by biomarkers and carriers across membrane barriers; and in terms of Protection, by loading of cargos, production of techniques, and MSC circulation. Contribution of exosomes to the formation of Synapses and modulation of their NeuroTransmitter Release are relevant in terms of Protection

The diagnosis of the neurodegenerative diseases depend on specific neuronal biomarkers. The blood and its fluid derivatives are very convenient for their identification [57]. In AD they do not include only the proteins typical of the disease, i.e. Aβ42/40, tau and others. Amphiphysin 1, specific for microRNAs and exosome components, has a role with neuronal adhesion molecules [60, 67–69]. Additional markers can be used to reveal physical conditions of AD patients [70, 71].

Widely approved approaches shown for AD are valid also for PD, others are more specific. Critical of UPS is α-synuclein associated with relevant components. Interestingly, the combination of α-synuclein with poly(ADP-ribose) has been shown to provide information about PD severity in neurons [72]. In contrast, the combination of the protein with EVs of possible anti-AD role was found to reveal cognitive impairment, a type of lesion relatively weak in PD patients [73]. Other analyses are of interest because, in addition to differences between PD and control, they include also AD markers. Thus, they can operate in the distinct diagnosis of the two diseases [74, 75].

In addition to AD and PD diagnoses, new results operate also with neuronal tauopathies. Unexpectedly, UPS of tau protein actions does not occur by a single but by a few events (Fig. 2), including fusion with intracellular vesicles together with traffic through the plasma membrane [50, 51, 62]. The multiple mechanisms of tau processing contribute to the heterogeneity of such molecule [50]. In addition, tau expression is regulated by a critical transcription factor necessary to control the level of the protein in various conditions [75]. Recent studies have identified changes of tau expression including formation of disulfide bridges. In addition to hyper-phosphorylation these changes, including its distribution among adjacent neurons, are relevant in AD pathology [63, 64]. Results that induce not only secretion but also spreading of tau by navigation of exosomes, are active for tauopathies and other related neurodegenerative diseases [65, 66].

### Inflammasomes and other UPS events

Inflammasomes are protein complexes containing enzymes, interleukins and ensuing inflammatory cascades, activated in the cell cytoplasm by infectious and/or non-infectious stimuli. UPS of immunoregulatory proteins trigger novel paradigms of effects. Together with exosomes they play key roles in inflammatory responses [48, 76, 77]. Such perspectives contribute to the state of immunology in many cells [76, 77]. In particular, stimulated lymphocytes of T and B type provide the immune system with finely tuned strategies to increase their crucial secretory functions [78]. Information about UPS of immunity is available especially in the central nervous system. Direct interactions by important EVs from microglia immune cells have been found to induce key roles in neuron-inflammatory responses [79]. Microglial cells also participated in the TNF-α-induced propagation of α-synuclein through neurons. Interest about such processes requires activation of endo-lysosomal exocytosis combined to stimulated senescence [80], largely dependent on exosome EVs [79].

Additional studies have revealed participation of UPS in new forms of disease. Closely linked to inflammation and immunity are events of heat shock proteins, distributed not within but outside the cells. By providing proteostasis and ATP, they play roles in cancer, neurodegeneration and other diseases [81, 82]. Moreover, kinase-mediated forms of UPS rescue misfolded proteins [83]. Long-tailed myosins, by interaction with other proteins, participate in the regulation of mast cell exocytosis [84]. Glypican-1, a form of heparin sulfate proteoglycans, drives unconventional secretion of FGF2, a survival cell factor [85]. APh, in addition to its canonical degradation upon fusion with lysosomes, induces wide forms of UPS revealed by markers such as Atg5 and BCNI [86, 87]. Another form of APh secretion prevents gut dysbiosis by excessive intestinal inflammation [88, 89]. I have already mentioned the case of GRSP55, a Golgi protein that, depending of its level of phosphorylation, acts in either conventional secretion or UPS [55]. Analyzed for its UPS, GRSP55 has been found to operate in a signaling axis with mTORC1 [89, 90] and to affect many relevant proteins such as huntingtin, tau, TDP43 and others, inducing results of variable strength [91]. Interestingly, this and other proteins of the Golgi apparatus participate in the generation and development of other diseases [89].

### Role of UPS in cancer

**Fig. 3:**
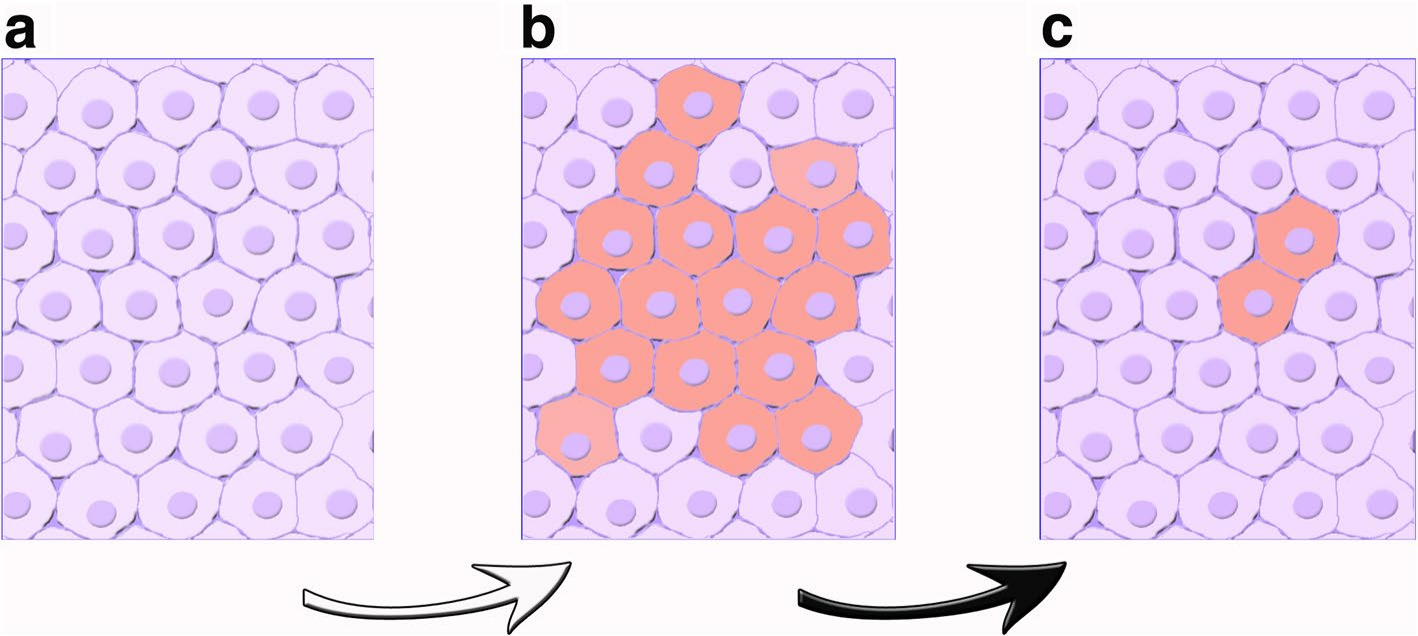
Role of exosomes in cancer cells. **a** Normal cells with adequate levels of exosomes, showing no cancer cells. Their growth occurs at normal rate (not shown). **b** Administration of high levels of exosomes (white arrow) can participate in cancer initiation and progression, dangerous also for the appearance of many other properties. **c** Administration of cancer growing cells together with exosomes loaded by therapeutic factors (black arrow): for example, adequate antibodies, chemotherapeutic drugs, therapeutic genes or other agents, inducing strong depression of cancer growth. The possible final step is elimination of cancer (not shown)

Various aspects occurring in neurodegenerative and other diseases are not entirely different from the UPS events occurring in cancer. Similarities of these processes with those concerning physiology of brain cells, have been reported recently [92]. On the other hand, properties of cancer revealed during the last decade have confirmed the coexistence of specific cells, now identified as cancer stem cells (CSCs), together with generally distributed mesenchymal stem cells (MSCs). Present evidence recognizes these similar types of cells as intense generators of exosome EVs, critical initiators and developer of cancers, involved in the formation of metastases, therapy resistance, and cancer relapse [30]. To sustain these typical activities, CSCs and MSCs induce intense UPS secretion, dealing with interactions between structures co-governing various operative mechanisms [30]. Cancer cells have been reported to generate specialized EVs of exosome nature, more numerous than those of normal cells. These observations suggest an important role of cancer cells, relevant in terms of generation and function of exosomes. (Fig. 3b) [30, 92]. Interestingly, such results have been reported to occur under intracellular control by APhs and their inhibitors [26, 31]. At present the innovative interactions between distinct mechanisms of UPS generation are of great importance for at least two reasons: the state of cancer development and their therapeutic approaches [30, 31]. The latter problem will be considered specifically in the next Section.

Various types of cancer reveal specific aspects of immunology. EVs influence biology of tumor cells by including distinct forms of their components, such as proteins, enzymes, and RNAs, thus forging tumor immunology [32]. Their task is to provide new angles of immune-oncology, involved in new strategies of cancer immunotherapies [33]. Brain tumors, such as aggressive gliomas and fatal cancers with dismal prognosis, include various UPS proteins involved in growth, proliferation and invasion. In other words, UPS machinery participates in the aggression and maintenance of brain tumors [34].

Among these cancers are those of breast, very aggressive against women in the whole world [35]. Their tumor microenvironment involves extracellular matrix together with active factors, such as cytokines, chemokines and exosome EVs, to orchestrate UPS tumor progression (Fig. 3b) [93]. Growth and development of presentations in niches govern metastatic colonizations, with ensuing vascular leakiness, angiogenesis, immunomodulation and chemoresistance together with fluid cancer. Exosomes, combined with APhs, govern recent advances about characterization and functional analysis resulting in diagnosis, prognosis and perspectives of therapy [93].

## Role of UPS in therapy

Therapy is a major tool of medical practice often connected to other activities (see, for example, [30, 31, 33, 59–61, 77, 93]). UPS therapy, illustrated in this Section, is based on organelles, mostly exosomes, activated by mechanisms and processes already illustrated. Exosome EVs are increased in many diseases and very abundant in cancers (Fig. 3a). The action, induced upon their loading by drugs or other factors, is very advantageous compared to analogous delivery systems, such as liposomes and polymeric nanoparticles [93, 94].

Properties of exosomes maintained by UPS depend on their extensive navigation and targeting, from their cells of origin to their specific target cells, and vice-versa. As already mentioned, the critical role of exosomes in medical interests and functions is largely integrated by MSCs that in cancer includes also the specific forms CSCs [30, 31]. In living cells, the immense relevance of exosomes is largely due to their intrinsic features, including low immunogenicity, biocompatibility, stability, and ability to cross barriers [93–95]. However, details of the exosome effects in UPS remain to be fully explained [93, 95].

### Complex therapy of neurodegenerative diseases

Among non-cancer diseases, AD is the one affecting the highest number of patients showing decline of memory, thought and behavior impairment, ultimately leading to death. Intense attempts of therapy, emerged initially from pre-clinical models, developed during the last decades. The present approach is based on the generation of Aβ-related factors. The present BACE-1 blocker studies are based on the identification of hundreds, or even thousands, of such possible drugs, analyzed by multiple distinct procedures. So far, however, the ensuing, more and more attempts have remained of unproven efficacy [96]. The present non-conclusive state of AD BACE-1 therapy has been confirmed this year, when all attempts of clinical trial have failed [97]. The analysis of important drugs and of their results continues.

Another form of AD therapy deals with human EVs, based on vesicle engineering dependent on good manufacturing practice (GMP) developed from conditioned media [98, 99]. Several properties of recent studies have been highly relevant. Among them are the selection of materials, the multiplicity of the programmed experimental conditions, and the quality of the assays employed for the evaluation of developments and results. So far, promising results have been obtained with MSCs and, even better, with MSC-EVs, increasingly considered as bio-therapeutic agents for a variety of different diseases. Combinations of engineered and natural approaches have been studied. The conditions investigated are still numerous. In perspectives, they are considered potentially valid for efficient therapies, especially for those active in regenerative medicine [98, 99].

The present state of PD, the other major neurodegenerative disease, is profoundly different from that of AD. The classical therapy of the disease, aimed to the protection of dopaminergic neurons, is still very active and increases considerably the natural length of the disease. The innovative therapy is based on drugs of various effects interesting for possible advantages. Here I will start with the rotigotine patch, a dopamine agonist, advantageous for its effective dermal administration [100]. Rotigotine induces no inconveniences and adverse effects compared to conventional drugs of other origin also employed, including gabapentinoid and pregabalin [101]. In addition, another UPS protein, a-synuclein, has been identified as a putative biomarker of neurodegeneration associated with PD. Molecules that target a-synuclein may thus serve as therapeutics, possible advantages to present options such as levodopa and dopamine agonists [102].

### Therapy for various types of cancer

The processes discussed here are based on UPS and their EVs, especially exosomes involved in cancer initiation and progression (Fig. 3b). Among exosomes, some are closely related to properties positive for many cancers, dependent on immunity, angiogenesis, pre-metastasis microenvironment, chemoresistance, energy metabolism and others. These properties or their block operate not only in normal physiology but also in the development of various cancer therapies, involving block of generation, secretion, and elimination of circulating exosomes, with additional use of anti-cancer vaccines. Exosomes can be loaded by chemotherapeutic drugs, therapeutic genes, and other agents addressed to target cancer cells (Fig. 3b-c) [103–105]. Pharmacological and operational activities remain integrated, reviewed by early phase of clinical trials [105]. Moreover, exosomes loaded with adequate drugs can be employed not only against cancer processes including metastases, but also in other conditions such as viral practice transmission and neurodegenerative diseases. In all these cases inhibition of EV secretion may delay therapeutic progression [106].

Exosomes have integrated many properties of UPS including their pass through natural barriers, extending the time and space of their circulation. Recent innovative changes have induced dramatic increases of vesicles in cancer treatments based on therapeutic cargos, loading methods, targeting strategy, and other perspectives of cancer therapy [103, 104]. In addition to proteins, present interest deals also with other luminal EV components. For example, three miRNAs with known oncogenic role, miR-21, miR-141, and miR-45, have been investigated in terms of drug therapy, focusing on gene regulation and intercellular communication [106].

### Activity of a few types of cancers

Here I intend to summarize a few types of cancer therapy. In agreement with concepts already established [78], various types of lymphocyte tumors, Burkitt lymphoma and human myeloid leukemic cells, have started to be considered for advanced therapies. The innate ability of passive exosomes was distinguished from the targeting capability of EVs, each engineered with a monoclonal antibody, enhancing their internalization into target cancer cells. In particular, specific therapeutic targeting of EVs was identified with respect to other inactive vesicles [107]. Analogously, immune responses of T cells have become the focus of immunotherapy against neoplastic diseases, especially of hematological malignancies, such as acute/chronic leukemia and multiple myeloma. Moreover, unconventional T cells could be useful in the treatment of specific forms of hematological neoplasms and in the development of an effective anticancer vaccine therapy [108, 109]. Analogously, EVs from MSCs were shown to promote neurodegeneration.. Such strategy may provide an impetus for improving the efficacy of MSC vesicles in reversing the process of neurodegeneration [110].

For strong cancers, including breast and gastric cancers expressing epidermal growth factor receptor 2 (HER2), various approaches have been developed. The action of the well-known anti-HER2 antibody trastuzumab has been combined to the cytotoxicity of chemotherapeutic drugs, with interesting improvements expected also for future directions [111, 112]. However, comparison between two previously approved chemotherapeutic drugs, deruxetan and emtansin, administered together with the trastuzumab antibody, has failed to reveal any difference and thus any economical advantage [113].

## Conclusions

A task of the present review has been to expand knowledge about UPS. Some information was already available, concerning, for example, its role in unconventional protein secretion. In addition, UPS was known to operate on specific types of diseases, which however had been mostly investigated separately. During the last several years, two general reviews about UPS diseases had appeared [22, 23], which however they are largely distinct from the present review. Specifically, therefore, a general view about numerous UPS-dependent diseases was not available. Based on these considerations I thought that time had come to promote a general UPS review of my type.

The present review was organized in three Sections. The first provided solid information about USPs operative under healthy conditions in many cells. During the following, knowledge about healthy processes reinforced the understanding of the processes occurring in UPS-dependent diseases. Even more exciting has been the presentation about therapies. Here interest focused primarily on exosomes, small EVs employed as such and upon their loading with aggressive factors against cancer. The attempts and the positive results obtained in the present review have been connected to pharmacological and clinical work, useful to explain the nature and the results of the UPS-dependent therapies.

The report in this review of many papers about UPSs was related to the time of their appearance in the literature. Among the data I have reported, only some had been published several years ago. The large majority appeared during the last 3 years, and a significant fraction in the present year, 2022. Many of such recent publications have introduced or reinforced new data and ideas, relevant for the diseases. Together with these improvements, however, recent changes may still need corrections, while a few additional data remain to be discovered. In other words, the need of their further improvements cannot be excluded. I hope that at least some of these hypothetical improvements, which remain to be identified, will become included in the literature of the near future, progressively solving possible limitations of the present review.

## Data Availability

The data of this review will be available to all scientists with specific interests.

## Abbreviations

AD: Alzheimer’s Disease
APh: AutoPhagy
cdc42, RhoA, Rock: Small G proteins
CSC and MSC: Cancer and mesenchymal stem cells
ER: Endoplasmic Reticulum
ESCRT: Endosomal Sorting Complexes Required for Transport proteins
EV: Extracellular Vesicle
GC: Golgi Complex
MVB: MultiVesicular Body
PD: Parkinson’s Disease
